# Chemo-Immunotherapy with Oxaliplatin and Interleukin-7 Inhibits Colon Cancer Metastasis in Mice

**DOI:** 10.1371/journal.pone.0085789

**Published:** 2014-01-21

**Authors:** Hong-Feng Gou, Juan Huang, Hua-Shan Shi, Xin-chuan Chen, Yong-Sheng Wang

**Affiliations:** 1 Department of Abdominal Cancer, Cancer Center, the State Key Laboratory of Biotherapy, West China Hospital, West China Medical School, Sichuan University, Chengdu, China; 2 Department of Head and Neck Cancer, Cancer Center, the State Key Laboratory of Biotherapy, West China Hospital, West China Medical School, Sichuan University, Chengdu, China; 3 Department of Hematology, West China Hospital, West China Medical School, Sichuan University, Chengdu, China; 4 Department of Thoracic Cancer, Cancer Center, the State Key Laboratory of Biotherapy, West China Hospital, West China Medical School, Sichuan University, Chengdu, China; Istituto Superiore di Sanità, Italy

## Abstract

Combination of immunotherapy and chemotherapy has shown promise for cancer. Interleukin-7 (IL-7) can potentially enhance immune responses against tumor, while oxaliplatin (OXP), a platinum-based drug, can promote a favorable immune microenvironment and stimulate anticancer immune responses. We evaluated the anti-tumor activity of IL-7 combining OXP against a murine colon carcinoma in vitro and in vivo and studied the tumor immune microenvironment to investigate whether the combined treatment affects on the local immune cell populations. Utilizing lung and abdomen metastasis models by inoculation of CT26 mice colon cancer cells, we evaluated the anti-tumor efficacy of combining IL-7 and OXP in mice models. Tumor immune microenvironment was evaluated by flow cytometric analysis and immunohistochemical staining. Our study showed that the in vivo administration of IL-7 combined with OXP markedly inhibited the growth of tumors in lung and abdomen metastasis models of colon cancer. IL-7 alone had no effect on tumor growth in mice and IL-7 did not alter cell sensitivity to OXP in culture. The antitumor effect of combining IL-7 and OXP correlated with a marked increase in the number of tumor-infiltrating activated CD8+ T cells and a marked decrease in the number of regulatory T (Treg) cells in spleen. Our data suggest that OXP plus IL-7 treatment inhibits tumor cell growth by immunoregulation rather than direct cytotoxicity. Our findings justify further evaluation of combining IL-7 and chemotherapy as a novel experimental cancer therapy.

## Introduction

Oxaliplatin (OXP), which is commonly used in colorectal cancer, has been recently found to increase the immunogenicity of cancer cells and induce immunogenic cell death [1]. Additionally, it has been found that OXP can enhance the immune response against tumors by decreasing regulatory/suppressor cells: regulatory T (Treg) cells and myeloid-derived suppressor cells (MDSCs) and increasing the ratio of cytotoxic CD8+T lymphocytes (effector cells) versus immunosuppressive cell populations in the tumor microenviroment [2], [3], [4].

Interleukin-7 (IL-7) is one of the Interleukin-2 (IL-2)-related cytokines that share and signal through the γ-chain to affect T cell proliferation,development and homeostasis[5], [6], [7], [8]. IL-7 is produced by a variety of stromal cells in the thymus and bone marrow, and also by vascular endothelial cells, intestinal epithelium, keratinocytes, and follicular dendritic cells [9], [10]. IL-7 represents a potential treatment to enhance T-cell reconstitution and vaccine efficacy as it has distinct actions on different subsets of T-cells[11]. IL-7 also promotes antigen-specific T cell cytolytic activity [12], [13], [14]. IL-7 was consistently as effective as IL-2 in maintaining T cells [13], [14]. For example, it has been demonstrated that tumor cell lines transfected with the IL-7 gene reduced T-cell–dependent tumorigenicity in murine models [15], [16]. Similarly, the local or systemic administration of IL-7 has anti-tumor effects by enhancing immune response against tumors [17], [18], [19], [20], [21], especially when combined with other immune treatment. The ability to enhance immune response against malignancies of IL-7 has major implications for immunotherapy in the treatment of tumors.

The combination of chemotherapy with immune response modifiers such as interleukin 2 (IL-2) or interferon-α (IFN-α), referred to as chemo-immunotherapy, has shown promising anti-tumor activity to melanoma [22], [23]. Cytotoxic chemotherapeutic agents had traditionally been considered to have immunosuppressive side effects and be detrimental to anti-tumor immunity because of their nonspecific cytostatic and cytotoxic effects. However, there is accumulating evidence showing that under certain conditions some of these agents can affect the tumor immunological microenviroment and stimulate anticancer immune responses [3], [4], [24], [25], [26]. It is a rational development to combine these immuno-stimulatory cytotoxic chemotherapeutic agents with immune response modifiers.

Based on the above, we hypothesis that combination of IL-7 and OXP may increase their anti-tumor activity by inducing the expansion of T cells and blocking T cell inhibitory pathways in the tumor immuno-microenvironment. In our study, we evaluated the antitumor activity of IL-7 combined with OXP against a murine colon carcinoma in vitro and in vivo and examined the tumor immune microenvironment to investigate whether this combined treatment affects local immune cell populations. Our data show OXP plus IL-7 is significantly more effective than IL-7 or OXP alone in inhibiting tumor growth in vivo. Our data suggest that OXP plus IL-7 treatment inhibits tumor cell growth by immunoregulation rather than directly cytotoxicity.

## Materials and Methods

### Cell line

Colon carcinoma cell line CT26 was obtained from American Type Culture Collection (ATCC). Cells were routinely cultured as monolayer in 75-cm^2^ square tissue culture flasks in a humidified atmosphere containing 5% CO_2_ in air. The culture medium contained RPMI 1640 (Life Technologies, Bedford, MA) supplemented with 10% FBS, 100 U/ml penicillin. The cell line was mycoplasma free.

### Tumorigenesis model

Pathogen-free female BALB/c (6–8 weeks old) mice were purchased from Vital River Laboratory Animal Technology Co. Ltd, Beijing. The protocol was approved by the Animal Ethics Committee of Sichuan University. All animal experiments were performed under specific pathogen-free conditions in accordance with institutional guidelines.

Before the in vivo injection into mice, the cancer cells in the exponential growth phase were harvested, washed thrice with PBS, counted, and diluted in this solution before in vivo injection. To establish a pulmonary metastasis model, a total of 1×10^6^ CT26 colon cancer cells were injected into the tail vein of syngeneic BALB/c mice. To establish an abdominal metastasis model, a total of 1×10^6^ CT26 colon cancer cells were intraperitoneally injected. Tumor-bearing mice were then monitored for tumor development and progression.

On day 6 after post-tumor inoculation each model was randomized into 4 groups with 8 mice in each group for further administration as follows: group of control (Phosphate Buffer Solution, PBS), group of IL-7, group of OXP, and group of IL-7 combined with OXP. In the abdominal metastasis model OXP was given i.p. at a dose of 5 mg/kg every 3 days for 12 days. In the lung metastasis model, OXP was given i.v. at a dose of 5 mg/kg every 3 days for 12 days. IL-7 (5 ug/day) was administered i.p. daily for 12 days.

The animals were euthanized on day 13 after the first treatment. Metastatic tumor nodules in the subpleural regions of the lungs were counted under a dissecting microscope and weighted. Seeding metastatic tumor nodules in the abdominal cavity were counted by naked eyes and weighted.

### Reagents

Recombinant human IL-7 (IL-7) was purchased from Shanghai PrimeGene Bio-Tech Co., Ltd and diluted in sterile distilled water. OXP was purchased from Sanofi-Aventis (France) and diluted in sterile glucose. Fluorescent-conjugated flow cytometry anti-mouse antibodies (Abs) CD4, CD8, CD69, CD11b, CD11c, Gr-1, and isotype-matched antibodies were obtained from BD Pharmingen. F4/80 was obtained from BioLegend. Mouse Regulatory T Cell Staining Kit was obtained from eBioscience. Immunohistochemistry antibodies pairs for murine CD8 was purchased from ABGENT. Ki-67 was purchased from Novus Biologicals, and Apoptosis Detection Kit was purchased from (Promega Corp., Madison, WI).

### 3-(4, 5-Dimethylthiazol-2-yl)-2, 5-Diphenyltetrazolium Bromide Assay (MTT)

The 3-(4, 5-dimethylthiazol-2-yl)-2, 5-diphenyltetrazolium bromide 96-well plate assay was used to determine the relative amounts of cells in wells after exposure to various agents. The CT26 cells were plated at 1,000cells/well in 96-well plates, and then incubated for an additional 24 h. Five replicate wells were used in these assays. Next, different concentration gradients of IL-7(25 ng/ml, 50 ng/ml, 75 ng/ml, 100 ng/ml) and OXP (10 umol/l, 30 umol/l, 50 umol/l) were added into wells and incubated for 24 h, 48 h in a humidified atmosphere containing 5% CO_2_ at 37°C. All assays included appropriate controls. At the end of the incubation, viable cells were quantified using the 3-(4, 5-dimethylthiazol-2-yl)-2, 5-diphenyltetrazolium bromide (MTT). Briefly, 20 µl of the MTT stock solution (5 mg/ml) was added to each well of an assay. After 2 h of incubation, the medium was removed and the converted dye was solubilized with DMSO (150 UL/well). The absorbance of the converted dye was measured at a wavelength of 540 nm. The conversion to cell number was based on a standard curve. The percentage of cell survival (survival rate) was calculated by dividing the absorbance value of the treated sample by that of the untreated control within each group.

### Flow Cytometric Analysis

At the indicated time points, the tumors and spleens were harvested from the mice, minced into small fragments and mechanically dispersed in 3-5 ml cold RPMI medium. The tumors cells were digested in 1 mg/mL of collagenase IV (Sigma), 0.1 mg/mL of DNase (Sigma) in RPMI 1640 at 37°C for 1 h. The spleen cells were depleted of erythrocytes by osmoticlysis and adjusted to concentration of 1×10^5^ cell in 100 ul of PBS. After this, single-cell suspensions of tumor cells and splenocytes were stained for 30 min on ice with 1 ug of the following fluorochrome-labeled antibodies, CD4, CD8, CD69, CD11b, CD11c, F4/80, or matched isotypy control antibodies and then washed once with PBS. Intracellular staining for FoxP3 was performed using mouse regulatory T cell staining kit, according to the manufacturer's instructions. The cells were acquired on a FACSCablibur flow cytometer (BD Biosciences), and 10,000_20,000 gated events were collected. The collected data were analyzed by FlowJo software 7.6. In cytometric analysis, we gate data using the FSC and SSC bivariate plot, then draw a region around the cluster of lymphocytes and then gate the fluorescence histogram on them. This gate, in the FSC vs SSC view, once established, is then applied to the analysis of all subsequent samples. Three mice from each group were killed to evaluate immunocyte CD marker expression.

### Immunohistochemical staining

The excised tumors fixed in 10% formalin were embedded in paraffin and sectioned at 4 um thickness. The sections were utilized for analysis of expression of CD8, and Ki-67 by immunohistochemistry staining with relevant monoclonal antibody. Specimens were immunostained by standard labeled streptavidin-biotin protocol. Briefly, sections of 4 µm were mounted on silanized slides and allowed to dry overnight at 37°C. After deparaffinization and antigen retrieval, tissue sections were incubated with CD8 antibody (1∶250 dilution) and Ki-67 antinbody (1∶200 dilution) at 37°C for 1 h and then at 4°C for overnight. The sections were then incubated with biotinylated goat antimouse immunoglobulin G (Zymed Laboratories Inc, USA) and subsequently incubated with horseradish labeled streptavidin (Zymed Laboratories Inc, USA). 3, 3′-diaminobenzidine was used as chromogen and hematoxylin as counterstained. Three mice per group were analyzed.

### Evaluation of immunohistochemical staining

The degree of CD8+ infiltration was observed in more than 10 independent high-power (×200) microscopic fields for each tissue sample. The five microscopic fields with the most abundant distribution were selected within the cancer cell nest. The average number of CD8+ T cells was counted in the five microscopic fields [27]. Determination of proliferative activity by Ki-67 imunohistochemistry was performed quantitatively by counting immunoreactive tumor cells in the most intense staining areas. In each sample, a minimum of 1000 tumor cells were scored in the microscopic areas showing the highest degree of immunostaining, and the results were expressed as the percentage of positive cells[28].

### Terminal deoxynucleotidyl transferase dUTP nick end labeling (TUNEL) Assay

The sections, the same as those used for immunochemistry analysis, were utilized for in situ analysis of apoptosis by TUNEL. The TUNEL staining was performed with an in situ apoptotic cell detection kit following the manufacturer's protocol. The slides were observed under an Olympus fluorescence microscope attached to a charge-coupled device camera. The images were acquired under ×10 and ×40 objectives using the Image-Pro software. Apoptotic index was determined by calculating the average number of nuclei apoptotic cells in 5 high-power microscopic fields (×400) selected from a central region in viable tumor areas, avoiding areas containing necrosis[29].

### Statistical Analysis

PRISM version 5.0 (GraphPad Software) was used for drawing graphs and statistical analysis. Results are reported as mean ± standard error (SE). Dual comparisons were made using the unpaired Student *t* test. *P*<0.05 was considered to be statistically significant.

## Results

### *In vitro* effect of OXP plus IL-7 on the growth of CT26 cells

**Figure 1:**
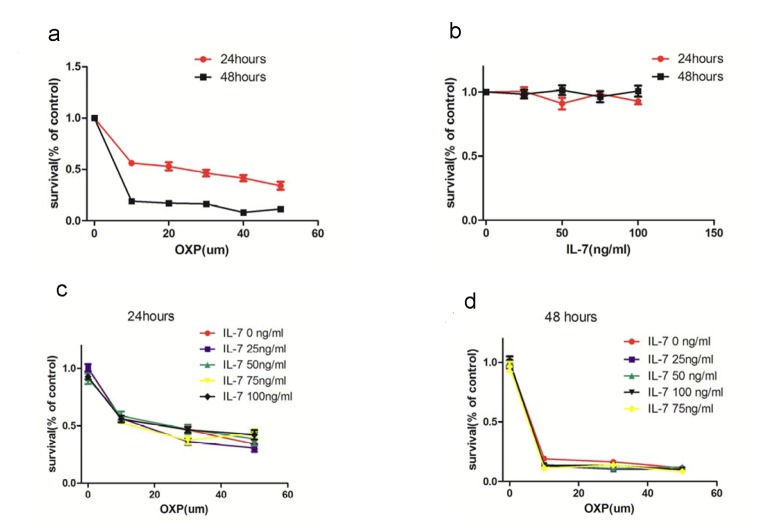
In vitro effect of OXP plus IL-7 on the growth of CT26 cells. CT26 cells were cultured in vitro with IL-7 (25 ng/mL, 50 ng/mL, 75 ng/mL, 100 ng/mL) alone or in combination with OXP at three concentrations from 10 µmol/mL to 50 µmol/mL and the relative numbers of viable cells were assessed 24, 48 hours later respectively. Each bar represents the mean ± S.E. (n = 5). a. OXP induced the expected concentration-dependent cell growth inhibition. b. IL-7 alone had no effect on CT26 cell growth in vitro. The rate of tumor cell growth was not significantly different at any dose level of IL-7 from those observed in control cultures. c and d. The addition of IL-7 did not significantly alter CT26 cell growth or their sensitivity to OXP.

To determine whether IL-7 directly affects the growth of CT26 cells or enhances their sensitivity to OXP in vitro, the effect of IL-7 alone and of IL-7 plus OXP on CT26 cells was determined in culture. CT26 cells were cultured in vitro with IL-7 (25 ng/mL, 50 ng/mL, 75 ng/mL, 100 ng/mL) alone or in combination with OXP at three concentrations from 10 µmol/mL to 50 µmol/mL. The relative numbers of viable cells were then assessed 24, 48 hours later respectively. OXP induced the expected concentration-dependent cell growth inhibition. The rate of tumor cell growth was not significantly different at any dose level of IL-7 from those observed in control cultures. And the addition of IL-7 did not significantly alter CT26 cell growth or their sensitivity to OXP **(** **Fig. 1** **).**

### Combination with IL-7 increases the anti-tumor effect of OXP in vivo

To investigate whether IL-7 might enhance the anti-tumor efficacy of OXP in tumor bearing mice, two models were established. Murine CT26 colon cancer cells were injected into BALB/C mice i.v. to establish the lung metastasis model and i.p. to establish the abdominal metastasis model. The mice received the following treatments: PBS; IL-7 (5 ug/injection); OXP (5 mg/kg/injection); and IL-7 (5 ug/injection) combined with OXP (5 mg/kg/injection), according to the above-mentioned schedule. The mice were euthanized on day 13 after the first treatment.

**Figure 2:**
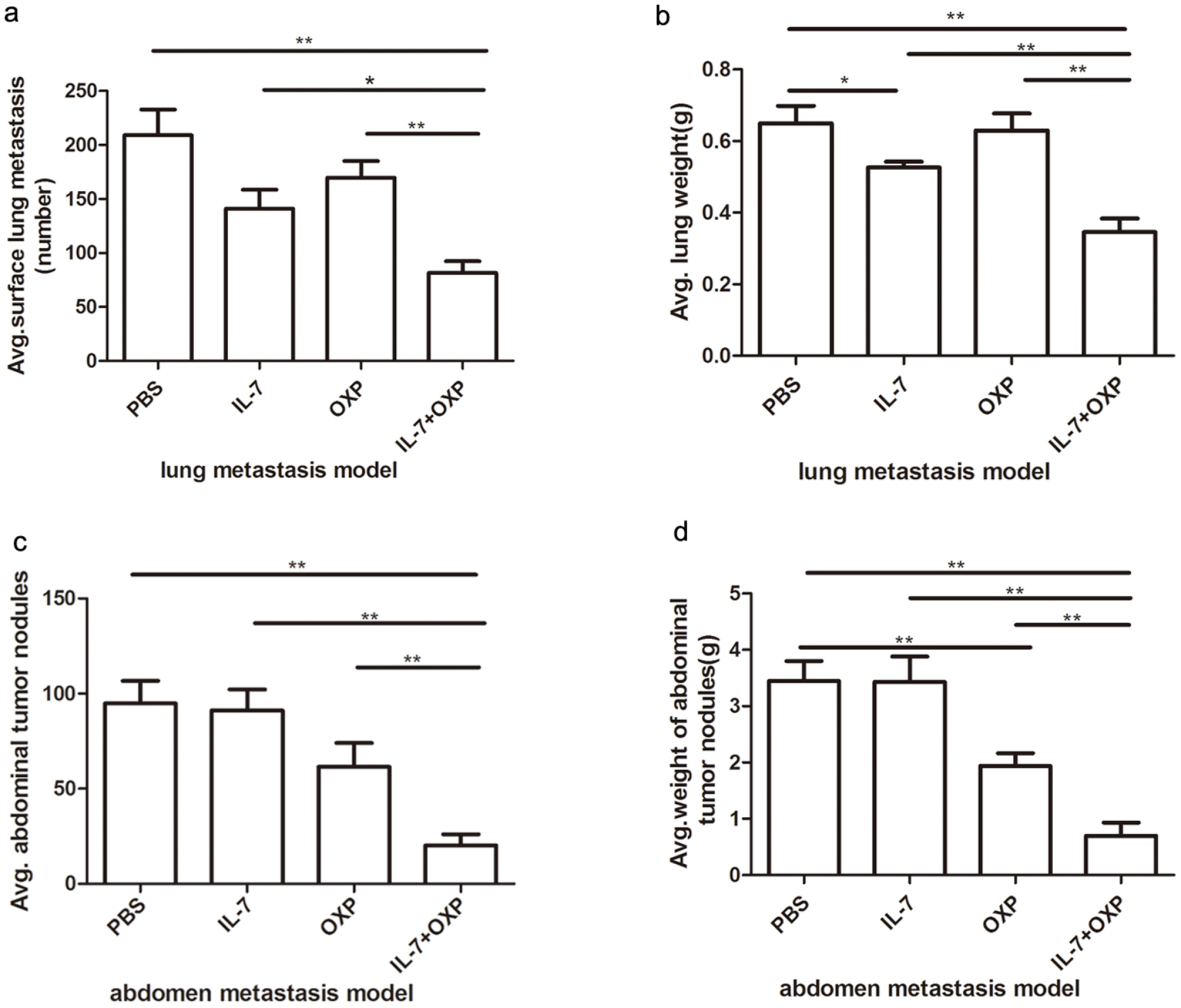
Combination with IL-7 increases the anti-tumor effect of OXP in vivo. On day 6 post-tumor inoculation each model was randomized into 4 groups with 8 mice in each group for further administration as follows: PBS, IL-7, OXP, IL-7 combined with OXP. Metastatic tumor nodules in the sub-pleural regions of the lungs were counted under a dissection microscope. The metastatic tumor nodules in the abdominal cavity were counted by naked eyes and weighted. The results were expressed as mean ± S.E. *P* values: *, *P*<0.05, **, *P*<0.01; a. Metastatic tumor nodules number in lung metastasis model. b. Weight of lungs in lung metastasis model. c. Metastatic tumor nodules number in abdomen metastasis model. d. Weight of tumor nodules in abdomen metastasis model. All of a, b, c, d showed IL-7, when given in conjunction with OXP, increased the anti-tumor effect in vivo compared to PBS, IL-7 and OXP groups respectively.The administration of IL-7 alone resulted in a significant reduction in the lung weights and a small, not statistically reduction in the final tumor volume compared to PBS in the lung metastasis model. The administration of OXP alone did not provide any anti-tumor effect compared to PBS in the lung metastasis model. In the abdominal implantation model, the administration of OXP alone resulted in a significant reduction in the weights of tumor nodules and a small, not statistically reduction in the number of tumor nodules in the abdominal cavity compared to PBS. The administration of IL-7 alone did not provide any anti-tumor effect compared to PBS in the abdominal implantation model.

In the lung metastasis model, the lungs were removed and weighed. The total numbers of lung metastatic nodules in the sub-pleural regions of the lungs were counted under the dissection scope. The results were expressed as mean ± S.E. The results clearly demonstrated that the combination of IL-7 and OXP achieves the best therapeutic effect, as measured by anti-tumor effect. As shown in **Fig. 2a** (n = 3), IL-7, when given in conjunction with OXP, had a greater than 2.55-fold, 1.73-fold, 2.07-fold reduction in the number of metastatic nodules on the lung compared to PBS,IL-7 and OXP groups respectively. The lungs were weighted. Proportionate decreases in lung weights were also observed. As shown in **Fig. 2b**, IL-7, when given in conjunction with OXP, had a greater than 1.88-fold, 1.52-fold, 1.82-fold reduction in the weights of the lungs compared to PBS, IL-7 and OXP groups respectively. Treatment with IL-7 alone resulted in a significant reduction in the lung weights and a small, not statistically reduction in the number of metastatic nodules on the lung compared to PBS. The administration of OXP alone did not provide any anti-tumor effect compared to PBS in the lung metastasis model.

In the abdominal implantation model, the tumor in the abdominal cavity were removed and weighed, and tumor nodules were counted and weighted. IL-7, when given in conjunction with OXP, increased the anti-tumor effect in vivo compared to PBS, IL-7 and OXP groups respectively (*P<*0.001, n = 6∼7). The administration of OXP alone resulted in a significant reduction in the weights of tumor nodules and a small, not statistically reduction in the number of tumor nodules in the abdominal cavity compared to PBS. The administration of IL-7 alone did not provide any anti-tumor effect compared to PBS in the abdominal implantation model. (**Fig. 2c, 2d**)

### Combination of OXP with IL-7 inhibited proliferation and induced apoptosis in mice colon tumor

**Figure 3:**
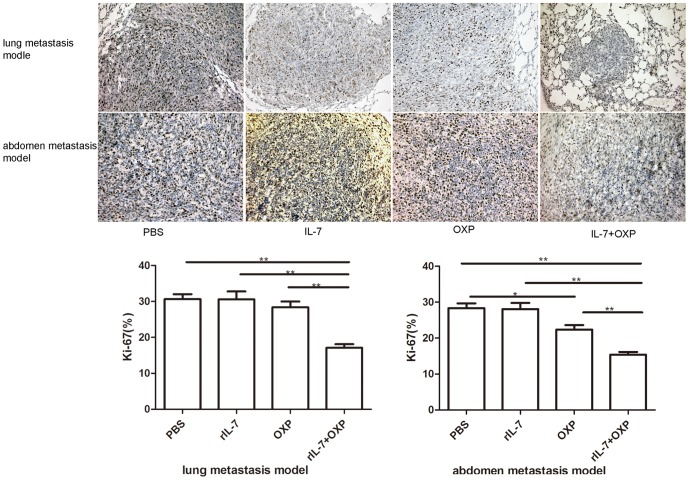
Combination of OXP with IL-7 inhibited proliferation in vivo. The resected tumors of each group were analyzed by immunohistochemical staining for the proliferation marker Ki-67. a. The representative slides of each treatment group are shown. Original magnification, ×200. b. The proliferative activity was evaluated by Ki-67 staining. In each sample, a minimum of 1000 tumor cells were scored in the microscopic areas showing the highest degree of immunostaining, and the results were expressed as the percentage of positive cells. The results were expressed as mean ± S.E. (n = 3). *P* values: IL-7+OXP compared with the other treatment groups, *, *P*<0.05, **, *P*<0.01. The combined treatment with OXP and IL-7 presented fewer Ki-67 positive cells, compared by IL-7 or OXP alone and control.

**Figure 4:**
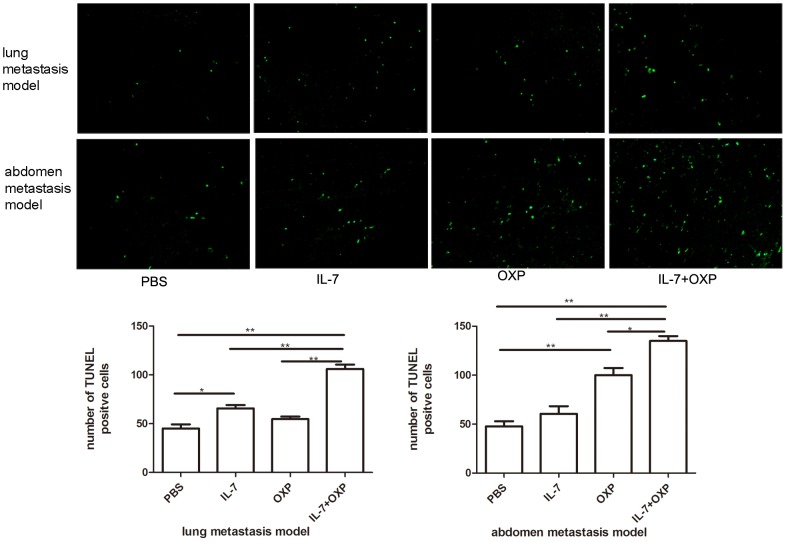
Assay of apoptosis induced by combination therapy by TUNEL. a. Paraffin sections were stained with the TUNEL analysis to detect apoptotic cells winthin CT26 tumor. The representative sections of each group are presented. Original magnification, ×200. b. Apoptotic index was determined by evaluating the average number of nuclei apoptotic cells in 5 high-power microscopic fields. The group of combination therapy showed the highest apoptotic index. Data represented apoptosic index were expressed as mean ± S.E. (n = 3). *P* values: IL-7+OXP compared with the other treatment groups, *, *P*<0.05, **, *P*<0.01.

We analyzed the number of proliferating cells using the proliferation marker Ki-67. The combined treatment with OXP and IL-7 presented fewer Ki-67 positive cells, compared with IL-7 or OXP alone and control (*P*<0.05) (**Fig. 3**). Furthermore, the TUNEL assay showed that the combined treatment with OXP and IL-7 induced significantly increased apoptosis of tumor cells (**Fig. 4**). The group receiving combination therapy showed the highest apoptotic index (*P*<0.01). These results suggest that combination of OXP with IL-7 significantly reduced cell proliferation and induced apoptosis *in vivo*.

### Combination of OXP with IL-7 induced significant activated T cells into the tumors

**Figure 5:**
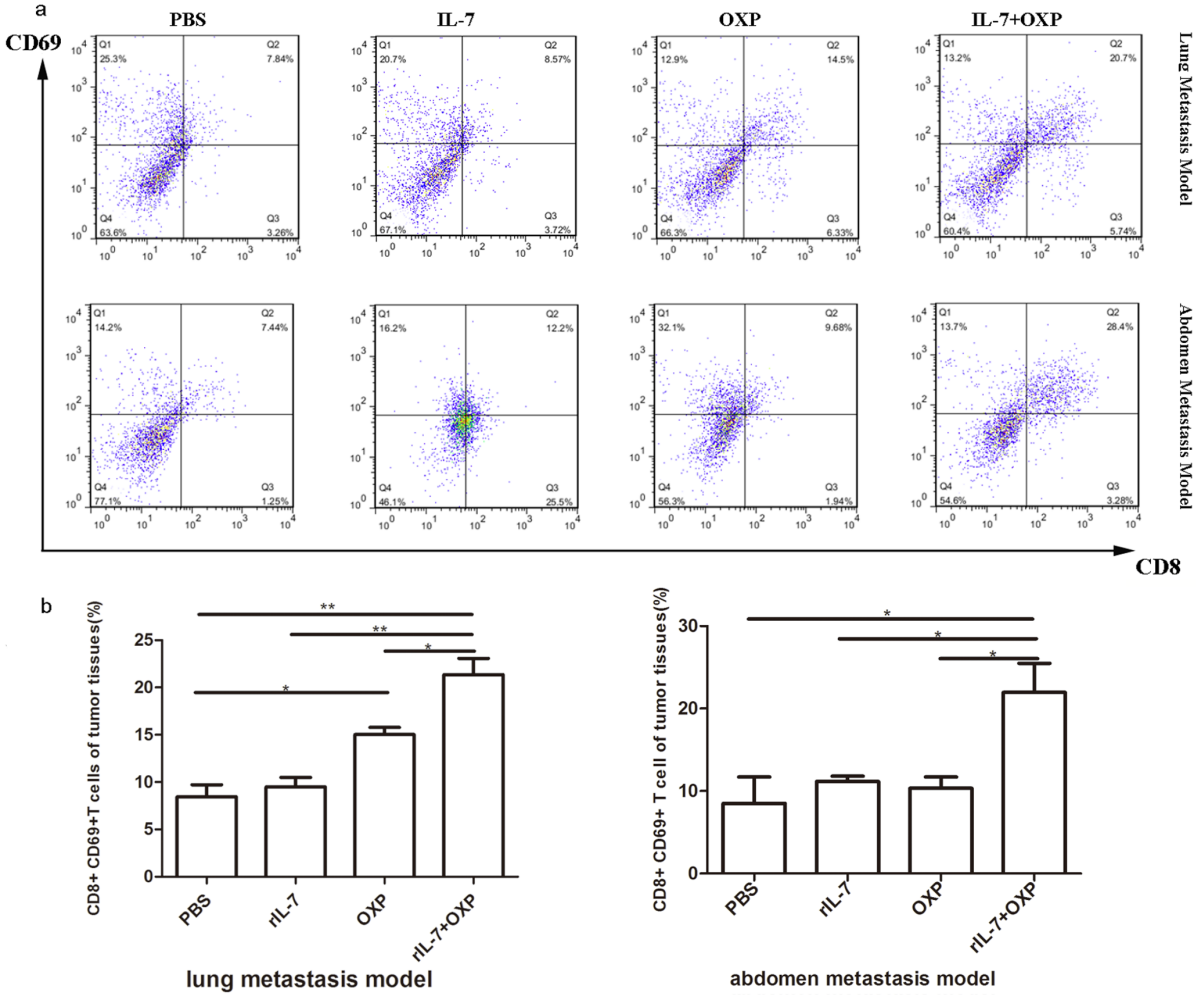
Combination of OXP with IL-7 induced significant activated T cells into the tumors. a. In cytometric analysis, we gate data using the FSC and SSC bivariate plot, then draw a region around the cluster of lymphocytes and then gate the fluorescence histogram on them. This gate, in the FSC vs SSC view, once established, is then applied to the analysis of all subsequent samples. Representative flow cytometry of each group was presented. b.IL-7 combined with OXP has significantly increased the CD8+/CD69+ positive cells in tumor compared with PBS controls and IL-7 or OXP alone lung model. The results were expressed as mean ± S.E. (n = 3). *P* values: IL-7+OXP compared with the other treatment groups, *, *P*<0.05, **, *P*<0.01.

**Figure 6:**
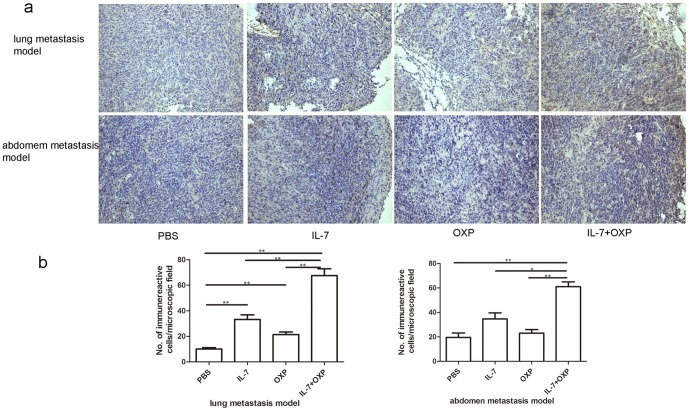
Immunohistochemical staining of CD8 in tumor tissue. a. The representative slides of immunohistochemical staining of CD8 each treatment group are shown. Original magnification, ×200. b. The degree of CD8+ infiltration was observed in more than 10 independent high-power (×200) microscopic fields for each tissue sample. The five microscopic fields with the most abundant distribution were selected within the cancer cell nest. The average number of CD8+ T cells was counted in the five microscopic fields. The results were expressed as mean ± S.E. (n = 3). *P* values: IL-7+OXP compared with the other treatment groups, *, *P*<0.05, **, *P*<0.01.

CD69 is a T-cell activation marker. We therefore determined whether IL-7, and/or OXP treatment enhanced the presence of activated CD8+ T cells (CD8+CD69+ population) in the two models murine colon tumors. In the lung metastasis model on day 13 after the first treatment, single-cell suspensions of tumor tissue from each group were analyzed for activated CD8+ T cells by flow cytometry. As shown in **Fig. 5**, IL-7 alone had no effect on CD8+CD69+ population. OXP alone has significantly increased the CD8 and CD69 positive cells in tumors compared with PBS controls and IL-7 too. IL-7 combined with OXP has significantly increased the CD8 and CD69 positive cells in tumors compared with PBS controls and IL-7 or OXP alone. An immunohistochemical study was performed on tumor samples obtained from each group of mice. The study was carried out to evaluate the amount of CD8+ T cells in tumor tissue. The results evealed that tumors from IL-7 and OXP combination-treated mice were heavily infiltrated with CD8+ T cells, compared with PBS, IL-7 or OXP alone treated mice (*P*<0.05) (**Fig. 6**). Similar results were observed in the abdominal implantation model (**Fig. 6**).

**Figure 7:**
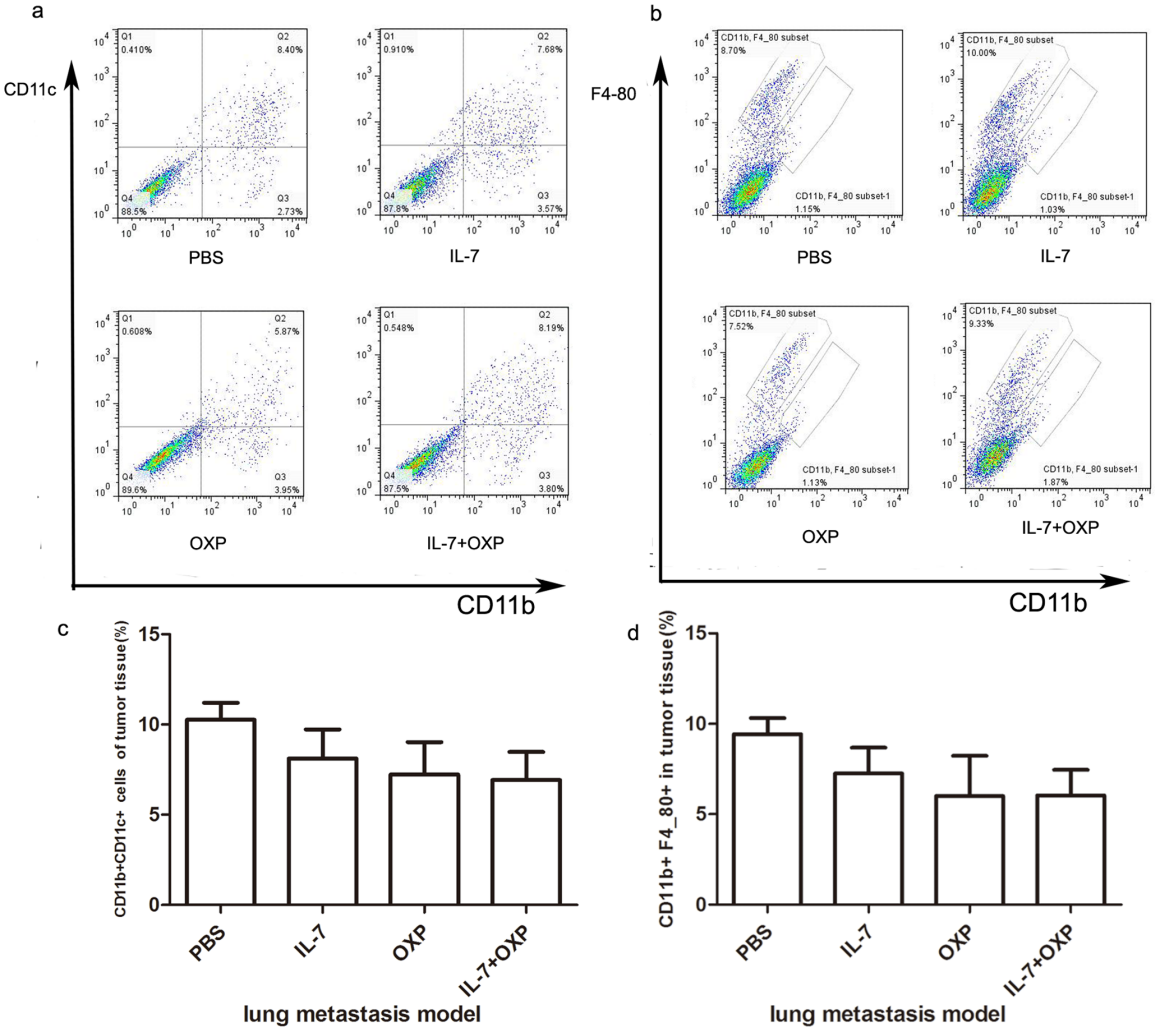
Combination of OXP with IL-7 had no effect the number of CD11b+F4/80 cells and DCs. a. and b. Representative flow cytometry of each group was presented. c. and d. IL-7 combined with OXP had no effect on the number of CD11b+F4/80+ cells and DCs in tumors compared with those from IL-7 alone, OXP alone, and PBS treated mice in the lung metastasis model.

Tumor-associated macrophages and dentritic cells (DCs) are major constituents of the leukocyte infiltrate in solid tumors. We also examined the number of macrophages in tumor by flow cytometry with F4/80 antibody and CD11b antibody and the number of tumor-infiltrating mature dentritic cells with CD11b antibody and CD11c antibody in the lung metastasis model. However, IL-7 and OXP treatments had no effect on the number of CD11b+F4/80+ cells and DCs in tumors compared with those from IL-7 alone, OXP alone, and PBS treated mice in the lung metastasis model (**Fig. 7**).

### Combination of OXP with IL-7 reduced Treg cells in spleen

**Figure 8:**
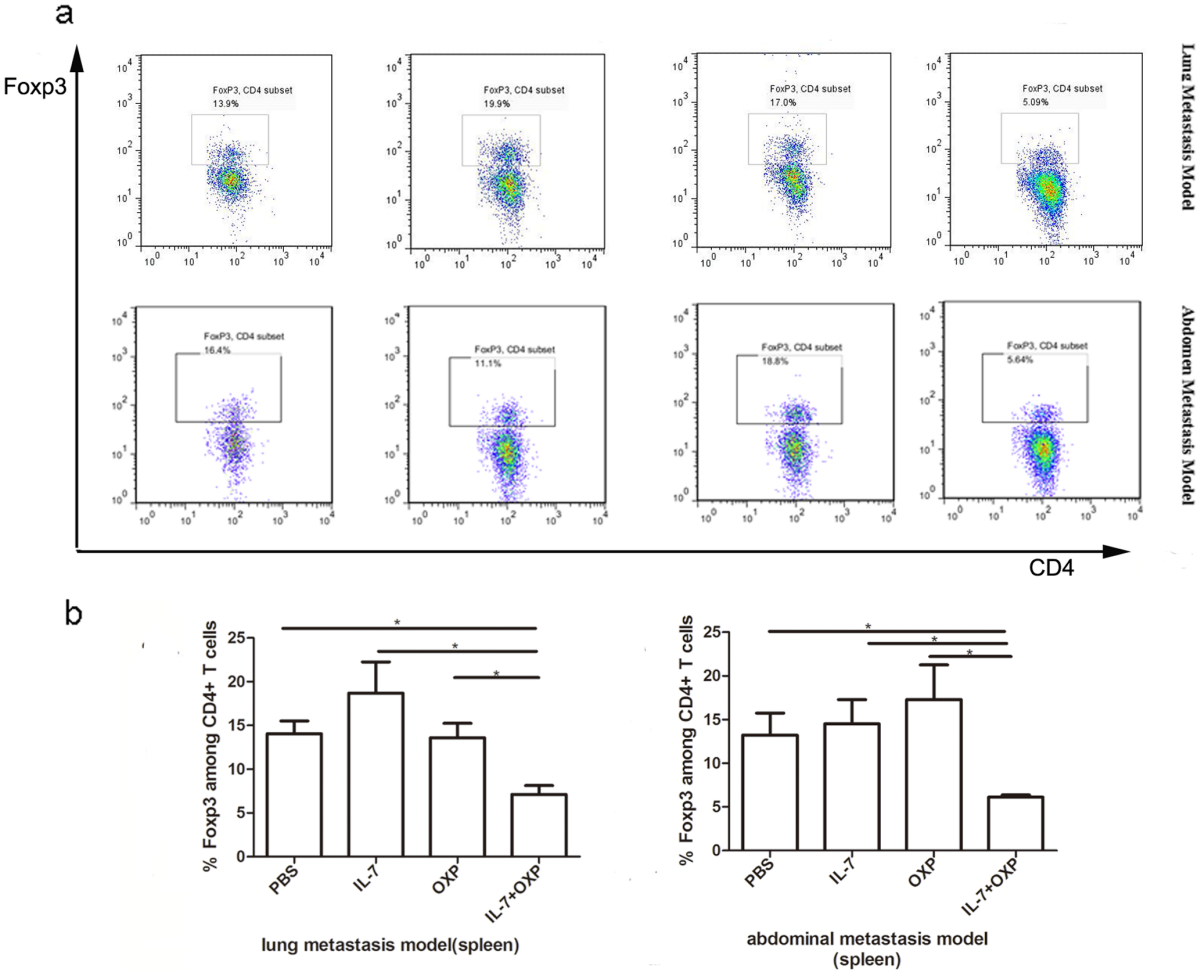
Combination of OXP with IL-7 reduces Treg cells in spleen. a. Representative flow cytometry of each group was presented. b. IL-7 combined with OXP has significantly reduced the FoxP3+ population in CD4+ T cells in spleen compared with PBS controls and IL-7 or OXP alone in both lung and abdominal metastasis models. The results were expressed as mean ± S.E. (n = 3). P values: IL-7+OXP compared with the other treatment groups, *, *P*<0.05, **, *P*<0.01.

We evaluated whether the IL-7 and OXP combined treatment had an effect on the immunosuppressive cell populations. Spleens from each group of animals were collected, and Treg populations (CD4+ FoxP3+ population) were analyzed by flow cytometry. In the lung metastasis model, we found that combination-treated CT26 colon tumor bearing mice had a lower percentage of Treg cells amongst CD4+ T cells in the spleen, in comparison with IL-7 alone, OXP alone, and control mice (Fig. 6)(*P*<0.05). Similar results were obtained in abdominal implantation model (*P*<0.05) (**Fig.8**).

**Figure 9:**
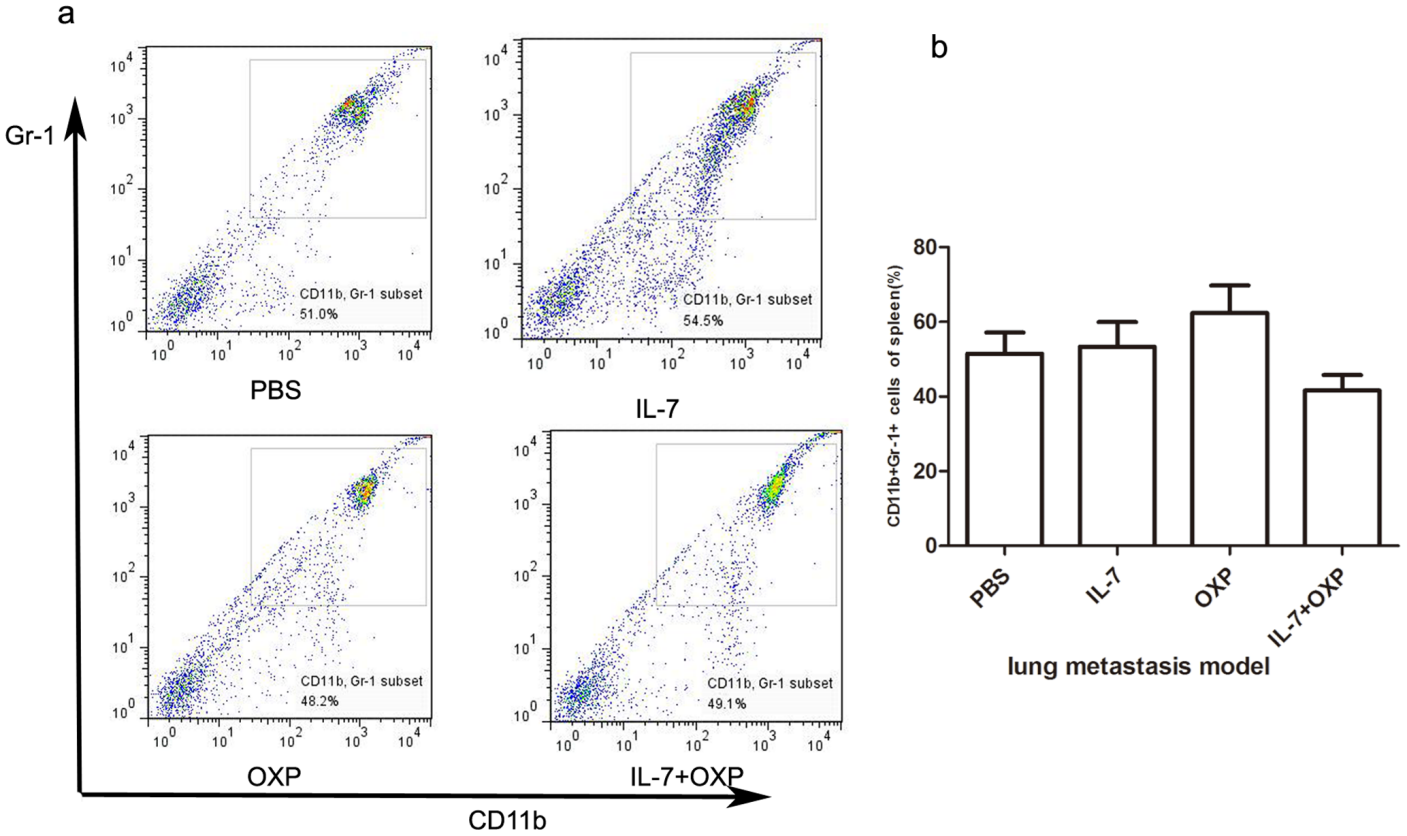
Combination of OXP with IL-7 had no effect on MDSCs. a. Representative flow cytometry of each group was presented. b. IL-7 and OXP did not significantly decrease the number of MDSC in spleen compared with that of IL-7 alone, OXP alone or control in the lung metastasis model.

MDSCs are heterogeneous populations of suppressor cells defined phenotypically in mice by their co-expression of CD11b and Gr-1[30], [31], [32]. We also examined the number of MDSCs in spleen in the lung metastasis model by flow cytometry with CD11b antibody and Gr-1 antibody considering their ability to suppress T-cell immune responses. IL-7 and OXP did not significantly decrease the number of MDSC in spleen compared with that of IL-7 alone, OXP alone or control in the lung metastasis model (**Fig. 9**).

## Discussion

The efficacy of the OXP plus IL-7 treatment against the highly invasive metastatic mouse CT26 colon adenocarcinoma was evaluated based on the rate of response in our experiment. Our data showed that when given in the same dose, OXP plus IL-7 is significantly more effective than IL-7 or OXP alone in inhibiting tumor growth. Our study shows that the OXP plus IL-7 combination treatment significantly inhibited tumor growth in murine models of colon cancer.

Our findings show that IL-7 alone had no effect on tumor growth in mice and IL-7 did not alter cell sensitivity to OXP in culture. Therefore, it is unlikely that IL-7 has a direct effect on the tumor. The flow cytometry data indicate that combination of OXP with IL-7 induced significant infiltration of activated T cells into the tumors and had a lower percentage of Treg cells in the spleen. These data suggested that OXP plus IL-7 treatment inhibited tumor cell growth by immunoregulation rather than direct cytotoxicity. The antitumor effects of OXP plus IL-7 were associated with increased percentages and number of activated CD8+ T cells. There were also associated with hampered development of Treg cells.

IL-7 can potentially enhance immune responses against tumor through a variety of mechanisms[8]. Many studies have found that IL-7 expands and maintains effective T cells in tumor-bearing mice. For example, IL-7 can enhance vaccine-mediated antitumor immunity by promoting CD8 expansion[21]. The markedly anti-tumor activity of administration of IL-7/HGFb in vivo correlated with a significant increase in the number of tumor-infiltrating CD4+ and CD8+T cells [18]. IL-7 synergizes with IL-12 or IL-21 to enhance anti-tumor immunity through the augmentation of cytotoxic T cells function[33], [34]. Enhanced generation of activated dendritic cells by IL-7 combined with other factors is another mechanism through which IL-7 may enhance antitumor immune response[18], [20]. Furthermore IL-7 can enhance anti-tumor immunity by antagonizing inhibitory networks: favoring Th17 differentiation, downregulating transforming growth factor (TGF)-β production, limiting TGF-β-associated signaling, and rendering the effector cells refractory to Treg cells[21], [35], [36], [37]. The ability of adjuvant IL-7 to antagonize inhibitory networks at the cellular and molecular level has major implications for immunotherapy in the treatment of tumors.

Most anti-tumor activities of IL-7 were found when it was combined with other therapeutic ways. Anti-tumor efficacy was nominal when IL-7 was used alone [16], [17], [18], [19], [20], [21], [35], [36], [38]. IL-7 alone has been found to have no effect on human colon carcinoma xenografts[38]. Chemotherapy agents may induce anti-tumor immunity and these findings open opportunities to integrate immunotherapies with chemotherapy. The anti-tumor activity of combining IL-7 and chemotherapy has not been studied. It has been reported that OXP can promote a favorable immune microenvironment and to stimulate anticancer immune responses [3], [4]. Exposure of calreticulin on the cell surface and release of HMGB1 have been proposed as key elements in the immunostimulatory properties of OXP [1].

OXP may enhance the immune response against tumors by tipping the balance between effector and regulatory/suppressor cells in favor of effector cells, as has been previously described for other chemotherapeutic drugs[2], [3]. A favorable balance between T-effector and T-suppressor cells has been associated with improved immune responses against tumors [2], [39]. The lack of efficacy of OXP alone in aggressive models in vivo has been described [3], [40]. Consistent with above observations, OXP alone had no antitumor activity in vivo in our study. However, OXP combined with IL-7 significantly inhibited tumor growth in murine models of colon cancer. The anti-tumor immune activity induced by OXP was enhanced by IL-7. When OXP was combined with IL-7, it may directly affect T-cell activation. A further explanation for the increased functional antitumor activity of combining OXP with IL-7 could involve suppression of the development of Treg cells, thereby retarding tumor development and growth. In our study, we did not find that IL-7 and OXP have effect on DC and macrophage.

A similar T cell effect was reported in several phase I clinical trials with minimal toxicities in which IL-7 was administered subcutaneously in the patients of cancer [41], [42], [43] and HIV infection [44], [45]. The limited toxicity of IL-7, as opposed to other cytokines such as IL-2, highlights the potential of this cytokine as a therapeutic agent [41], [42].

In conclusion, our findings show that the addition of IL-7 along with OXP can substantially increase antitumor immunity. However, the best way of integrating immunotherapy with chemotherapy is unclear. The relative importance and crucial nature of the effect remains to be explored. Further studies extending the length of IL-7 therapy, combining IL-7 with other chemotherapy agents will help elucidate the most effective use of this cytokine.
